# Cannabinoid Receptor 2 Protects against Acute Experimental Sepsis in Mice

**DOI:** 10.1155/2013/741303

**Published:** 2013-05-28

**Authors:** Huan Gui, Yang Sun, Zhu-Min Luo, Ding-Feng Su, Sheng-Ming Dai, Xia Liu

**Affiliations:** ^1^Department of Pharmacology, School of Pharmacy, Second Military Medical University, Shanghai 200433, China; ^2^Department of Rheumatology & Immunology, Changhai Hospital, Second Military Medical University, Shanghai 200433, China

## Abstract

The systemic inflammatory response syndrome can be self-limited or can progress to severe sepsis and septic shock. Despite significant advances in the understanding of the molecular and cellular mechanisms of septic shock, it is still one of the most frequent and serious problems confronting clinicians in the treatments. And the effects of cannabinoid receptor 2 (CB2R) on the sepsis still remain undefined. The present study was aimed to explore the role and mechanism of CB2R in acute sepsis model of mice. Here, we found that mice were more vulnerable for lipopolysaccharide- (LPS-) induced death and inflammation after CB2R deletion (CB2R^−/−^). CB2R agonist, GW405833, could significantly extend the survival rate and decrease serum proinflammatory cytokines in LPS-treated mice. GW405833 dose-dependently inhibits proinflammatory cytokines release in splenocytes and peritoneal macrophages as well as splenocytes proliferation, and these effects were partly abolished in CB2R^−/−^ splenocytes but completely abolished in CB2R^−/−^ peritoneal macrophages. Further studies showed that GW405833 inhibits LPS-induced phosphorylation of ERK1/2 and STAT3 and blocks I**κ**B**α** degradation and NF-**κ**B p65 nuclear translocation in macrophages. All data together showed that CB2R provides a protection and is a potential therapeutic target for the sepsis.

## 1. Introduction

The systemic inflammatory response syndrome can be self-limited or can progress to severe sepsis and septic shock. Pathogens or their products, such as lipopolysaccharide (LPS), play an important role in the process [1]. Upon LPS/Toll-like receptor 4 (TLR4) activation, immune cells can produce proinflammatory cytokines, such as tumor necrosis factor-*α* (TNF-*α*), interleukin-6 (IL-6), and high mobility group box-1 (HMGB1), overwhelming production of which might result in immunological and inflammatory diseases. One of the most severe examples is septic shock [2]. Despite significant advances in the understanding of the molecular and cellular mechanisms of septic shock, it is still one of the most frequent and serious problems confronting clinicians in the managements [3].

Identification and characterization of the negative regulator of LPS/TLR signaling attracts much attention in recent years. Some intracellular negative regulators such as interleukin-1 receptor-associated kinase-M (IRAK-M), suppressor of cytokine signaling 1 (SOCS1), A20, SHIP1, mixed-lineage kinase 4 (MLK4), Dok1/2, and RP105 have been identified to inhibit TLR4 signaling [4–8]. Recently, the effects of both synthetic and endogenous cannabinoids upon the immune system have acquired a great interest. There are two well-characterized cannabinoid receptors (CBR). Cannabinoid receptor 1 (CB1R) expresses primarily in central nervous system, which is associated with the psychoactive effects of cannabinoids. Cannabinoid receptor 2 (CB2R) expresses primarily by immune cells, which mainly mediates anti-inflammatory actions [9, 10]. CB2R agonists have potential utility as anti-inflammatory drugs for the treatment of many disease conditions, such as multiple sclerosis, rheumatoid arthritis, and autoimmune uveoretinitis [11–13]. These findings further make the CB2R an attractive therapeutic target in sepsis or septic shock. However, in the experimental sepsis, there are conflicting results regarding the effects of CB2R activation. It is reported that CB2R knockout mice following cecal-ligation-and-puncture- (CLP-) induced sepsis had a higher mortality and CB2R agonist improved survival of wild-type mice [14]. While in another study, CB2R seems like a strong destroyer in the same sepsis model [15]. Cannabinoid antagonist AM 281 was reported to reduce mortality rate after CLP in rats [16], while very recently, Lehmann and his colleagues found that CB2R activation reduced intestinal leukocyte recruitment and inflammation in rat acute sepsis models [17]. These controversial results leave this issue ambiguous. The specific contribution of CB2R to sepsis needs to be further explored.

Macrophages are activated early in response to immune challenge and are major players in both innate and adaptive immunity. Human macrophage-specific silencing of HMGB1 ameliorates sepsis in a humanized mouse model [18]. Lymphocytes are also important to host defense against infection. Adult patients dying of sepsis-induced multiple organ failure were found to have lymphocyte depletion and apoptosis [19]. Antigen-dependent T-cell activation influences survival in a murine model of sepsis [20]. However, whether macrophages and lymphocytes are the cellular target of CB2R in sepsis and the corresponding molecular mechanisms still remains undefined.

1-(2,3-Dichlorobenzoyl)-5-methoxy-2-methyl-(2-(morpholin-4-yl)ethyl)-1H-indole (GW405833) is a selective CB2R agonist, which has been proved to elicit efficacious antihyperalgesic effects against neuropathic and inflammatory pain in rat model [21]. Whiteside and his colleagues using CB2R knockout mice proved that GW405833 produces an antihyperalgesic activity through CB2R [22]. Here, GW405833 was firstly used as a selective CB2R agonist to evaluate the role of CB2R in sepsis shock.

The present study was designed to investigate the possibility for CB2R to be a new therapy target for the treatment of sepsis in vivo and further clarify the cellular and molecular mechanisms in vitro.

## 2. Materials and Methods

### 2.1. Reagents

GW405833, LPS, and Concanavalin A (ConA) were purchased from Sigma-Aldrich (St. Louis, MO, USA). Dulbecco's modified Eagle medium (DMEM), fetal calf serum (FCS), and phosphate-buffered saline (PBS) free of Ca^2+^ and Mg^2+^ were obtained from Life Technologies (GIBCO, CA, USA). RPMI-1640 medium was purchased from Hyclone (Shanghai, China). ELISA kits for mouse IL-6 and TNF-*α* were obtained from R&D Systems (Minneapolis, MN, USA). ELISA kit for mouse HMGB1 was purchased from Westang Biological Technology Co., Ltd. (Shanghai, China). Lymphocyte separation medium was obtained from Dakewe Biological Technology Co., Ltd. (Shenzhen, China). Recombinant rabbit-polyclonal antibodies to nuclear factor-kappa B (NF-*κ*B) p65 and monoclonal antibodies to glyceraldehyde-phosphate dehydrogenase (GAPDH) were purchased from Santa Cruz Biotechnology (Santa Cruz, CA, USA). Primary antibodies against extracellular signal-regulated kinase 1/2 (ERK1/2), phospho-ERK1/2, signal transducer and activator of transcription 3 (STAT3), phospho-STAT3, and inhibitor-kappa B alpha (I*κ*B*α*) were purchased from Cell Signaling Technology (Boston, MA, USA). IRDye-conjugated donkey anti-rabbit IgG and goat anti-mouse IgG were purchased from Rockland Immunochemicals Inc. (Gilbertsville, PA, USA). Alexa Fluor 565-conjugated donkey anti-rabbit IgG was purchased from Life Technologies (Carlsbad, CA, USA).

### 2.2. Animals

Male C57BL/6J mice (8 weeks old) were obtained from SLRC Laboratory Animal Co., Ltd. (Shanghai, China). CB2R gene knockout (CB2R^−/−^) mice were purchased from Jackson Laboratory (Bar Harbor, Maine, USA) and expanded under specific pathogen-free conditions in Laboratory Animal Centre of Second Military Medical University. All animals were fed standard mouse chow and water freely and maintained under constant conditions (temperature: 20–25°C; humidity: 40%–60%; light/dark cycle: 12 h). All procedures were conducted in accordance with the university guideline and approved by Ethical Committee for Animal Care and the use of laboratory animals of Second Military Medical University.

### 2.3. Endotoxic Shock

Male CB2R^−/−^ mice (weigh 18–22 g) and the wild-type littermates (CB2R^+/+^) were challenged with LPS in saline (15 mg/kg) intraperitoneally. Survival rate was recorded every 1 h after LPS injection for 24 h. Male C57BL/6J mice (weigh 18–22 g) were injected with different doses of GW405833 intraperitoneally half an hour in advance and then challenged with LPS (30 mg/kg). Survival rate was recorded for 72 h.

### 2.4. Serum Samples Collection

For CB2R^−/−^ and CB2R^+/+^ mice, serum was obtained 3 hours after LPS (5 mg/kg) intraperitoneally injection. For C57BL/6J mice, serum was collected 6 hours after drug administration followed by LPS (5 mg/kg) injection.

### 2.5. ELISA

Serum level of IL-6, TNF-*α*, or HMGB1 was determined according to the manufacture's instruction. For splenocytes, 500 *μ*L/well cell suspension were seeded in 24-well plate, and ConA was added at a final concentration of 5 *μ*g/mL immediately followed by GW405833 treatment for 24 h. Samples were centrifuged at 1,2000 ×g for 5 min to obtain the supernatants. For peritoneal macrophages, LPS (1 ng/mL) with or without GW405833 were added into culture medium. Twenty-four hours later, the supernatants were collected. The concentrations of IL-6, TNF-*α*, and HMGB1 were determined using ELISA.

### 2.6. Preparation of Mixed Splenocytes

Mixed splenocytes were separated using lymphocytes separation medium (EZ-Sep Mouse 1X) under the manufacturer's introduction. Briefly, isolated spleens were grinded with a syringe piston and passed through 200-mesh nylon net to obtain homogeneous cells suspending in the lymphocytes separation medium. After centrifugation (800 ×g for 30 min), the layer of lymph cells was transferred into another new 15 mL centrifuge tubes, washed with PBS for three times and resuspended in complete RPMI 1640 medium containing 10% FCS, 100 U/mL of penicillin, 100 *μ*g/mL streptomycin, and 100 *μ*g/mL amphotericin B. The cells were counted and seeded in 96- or 24-well plate at about 1 × 10^7^/mL for subsequent procedure.

### 2.7. Culture of Mouse Peritoneal Macrophages

Mouse peritoneal macrophages were collected 3 days after intraperitoneal (*i.p.*) injection of sterilized broth culture (1 mL) to CB2R^−/−^ or CB2R^+/+^ mice as described previously [23]. The cells were washed twice with PBS, resuspended in DMEM containing 10% FCS, and seeded at a density of 1–3 × 10^6^/mL in 6-well or 24-well plates. Two–four hours later, the culture medium was replaced to remove the nonadherent cells and then incubated at 37°C in a humidified 5% CO_2_ atmosphere overnight for the subsequent procedures.

### 2.8. Splenocyte Proliferation Assay

Splenocytes were seeded into 96-well plate at 1 × 10^7^ cell/mL in 100 *μ*L complete RPMI containing ConA (5 *μ*g/mL) with/without GW405833 and cultured for 24 h in a humidified, 5% CO_2_ atmosphere at 37°C. Cell proliferation was measured as previously described using CCK-8 purchased from Dojindo Laboratories (Kumamoto, Japan) [24]. Briefly, 10 *μ*L of CCK-8 reagent was added to each well 4 h in advance and the absorbance at 450 nm was determined by ELISA plate reader (Multiskan MK3, Labsystems, Finland).

### 2.9. Immunofluorescence

Peritoneal macrophages were treated with vehicle or LPS (1 ng/mL) in the presence or absence of GW405833 (10 *μ*M) for 30 min and then fixed and penetrated with 4% paraformaldehyde containing 0.3% Triton X-100 for 15 min. Cells were blocked with 5% bovine serum albumin for 30 min and incubated with primary anti-p65 antibody for 2 h at room temperature followed by incubating with secondary Alexa Fluor 565-conjugated donkey anti-rabbit IgG for 1 h. At last cells were counterstained with 4′,6-diamidino-2-phenylindole (DAPI) for 3 min [25]. Photographs were taken using CKX41 inverted fluorescence microscope (Olympus, Japan).

### 2.10. Western Blot

Cultured peritoneal macrophages were lysed with lysis buffer. Protein concentrations were determined using the Bradford method (Bio-Rad). The lysates were fractionated by Tris-glycine buffered 10% sodium dodecyl sulfate-polyacrylamide gel electrophoresis [26], transferred onto nitrocellulose membranes, and incubated overnight at 4°C with antibodies against STAT3 (1/1,000 dilution), phospho-STAT3 (1/2,000 dilution), ERK1/2 (1/1,000 dilution), phospho-ERK1/2 (1/2,000 dilution), I*κ*B*α* (1/1,000 dilution), or GAPDH (1/10,000 dilution). After washing, membranes were incubated with IRDye-conjugated secondary antibodies (1/5,000 dilution) and then scanned using Odyssey Infrared Imaging System (LI-COR, USA).

### 2.11. Statistical Analysis

Data were analyzed using SPSS16.0 software. One-way analysis of variance (ANOVA) with Tukey's post-test for multiple comparisons was used to compare GW405833 treatment groups with vehicle group for levels of inflammatory factors in serum and supernatants. The Kaplan-Meier analysis was used to estimate survival rate. Log-rank testing was used to evaluate the equality of survival curves. *P* < 0.05 was considered statistically significant.

## 3. Results

### 3.1. CB2R Protects against LPS-Induced Shock and Proinflammatory Cytokines Production in Mice

**Figure 1:**
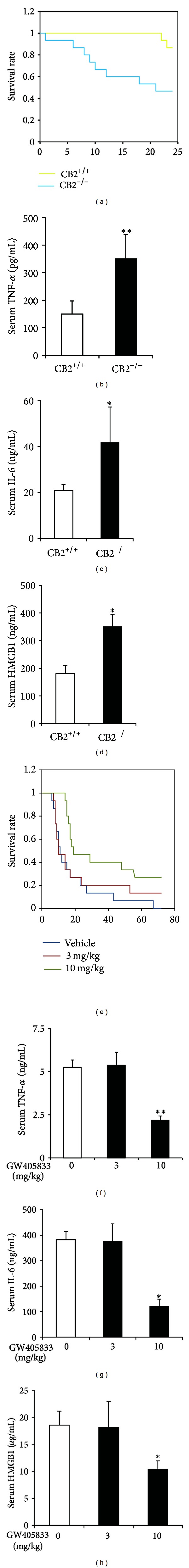
CB2R protects against LPS-induced shock and proinflammatory cytokines production in mice. (a) CB2R^+/+^ (*n* = 15) and CB2R^−/−^ (*n* = 15) mice were challenged with LPS (15 mg/kg, i.p.). Survival was assessed for 24 h. **P < 0.01. (b)–(d), ELISA detection of serum TNF-*α*, IL-6 and HMGB1 levels 3 h after LPS (5 mg/kg) challenge in CB2^+/+^ (*n* = 5) or CB2^−/−^ (*n* = 5) mice. Data are means ± SEM *P < 0.05, and **P < 0.01. (e) Observation of 72 h survival rate in C57BL/6 mice treated with LPS (30 mg/kg, i.p.) in the absence of presence of indicated doses of GW405833 (i.p.). Mice treated with vehicle as control. *n* = 15 per group and *P < 0.05 versus vehicle. (f)–(h) ELISA detection of serum TNF-*α*, IL-6, and HMGB1 levels 6 h after LPS (5 mg/kg, i.p.) challenge in the absence or presence of indicated doses of GW405833 (i.p.). *n* = 8 in vehicle, *n* = 5 in 3 mg/kg, *n* = 10 in 10 mg/kg, data are means ± SEM, and **P < 0.01 versus vehicle.

To investigate the role of CB2R in endotoxic shock, CB2R^+/+^ and CB2R^−/−^ mice were challenged with a lethal dose of LPS (15 mg/kg), and survival rate was observed. As shown in Figure 1(a), CB2R^+/+^ mice had a significantly higher survival rate compared with mice lacking CB2R (86.7% versus 46.7%, resp.). Consistent with this observation, serum levels of TNF-*α*, IL-6, and HMGBI were also significantly higher in the CB2R deficient mice as compared with the WT control group at 3 hours after 5 mg/kg of LPS administration (Figures 1(b)–1(d)), indicating that the genetic deletion of CB2R resulted in increased susceptibility to infection.

To evaluate the role of pharmacological activation of CB2R in sepsis, GW405833, a selective agonist of CB2R, was applied to the lethal dose of LPS-treated mice. After injection of 30 mg/kg LPS, mice began to die at 6 hours and no survivor was found at the end of 72 hours. Although 3 mg/kg GW405833 shows a little but not significant improvement in survival rate, 10 mg/kg GW405833 obviously increased the survival rate of mice (26.7%, Figure 1(e)). Consistently, 10 mg/kg GW405833 markedly decreased the serum levels of TNF-*α*, IL-6, and HMGBI at 6 hours after 5 mg/kg LPS injection (Figures 1(f)–1(h)).

### 3.2. GW405833 Inhibits ConA-Induced Splenocytes Proliferation Partially via CB2R Activation

**Figure 2:**
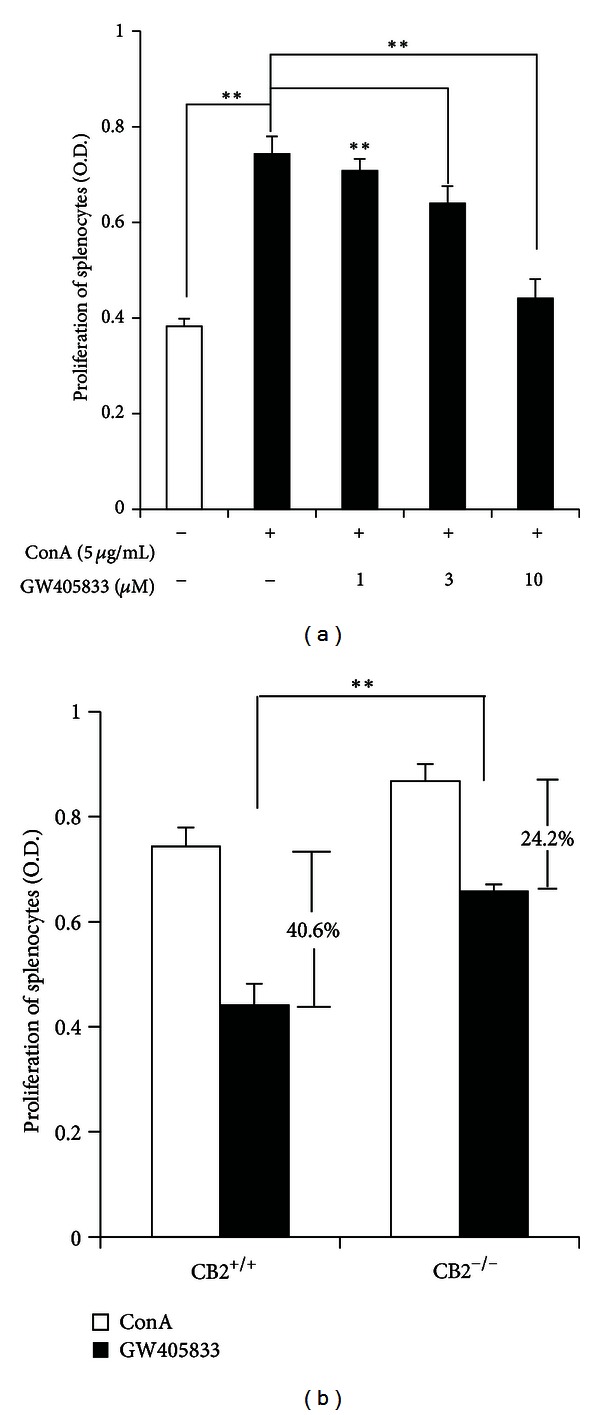
GW405833 inhibits ConA-induced splenocytes proliferation partially via CB2R activation. (a) Splenocytes isolated from CB2R^+/+^ mice were treated vehicle or ConA (5 *μ*g/mL) in the absence or presence of GW405833 (1–10 *μ*M) for 24 h. Splenocytes proliferation was detected by CCK-8. Data are means ± SD (*n* = 4) and **P < 0.01. (b) Splenocytes isolated from CB2R^+/+^ and CB2R^−/−^ mice were treated with ConA (5 *μ*g/mL) in the absence or presence of GW405833 (10 *μ*M) for 24 h. Splenocytes proliferation was detected by CCK-8. Data are means ± SD (*n* = 4) and **P < 0.01.

We studied the effect of GW405833 on splenocytes proliferation. ConA was used to stimulate T subtype of splenocytes to proliferate and differentiate. As shown in Figure 2(a), GW405833 dose-dependently inhibits Con-A-triggered splenocytes proliferation. When CB2R was deleted, the inhibitory effects were significantly attenuated. The inhibition ratio of GW405833 (10 *μ*M) on ConA-induced splenocytes proliferation was reduced from 40.6% to 24.2% (Figure 2(b)), which suggested that CB2R partially mediated the inhibitory role of GW405833 in splenocytes proliferation.

### 3.3. CB2R Partially Mediates the Inhibitory Effects of GW405833 on ConA-Induced TNF-*α* and IL-6 Production in Splenocytes

**Figure 3:**
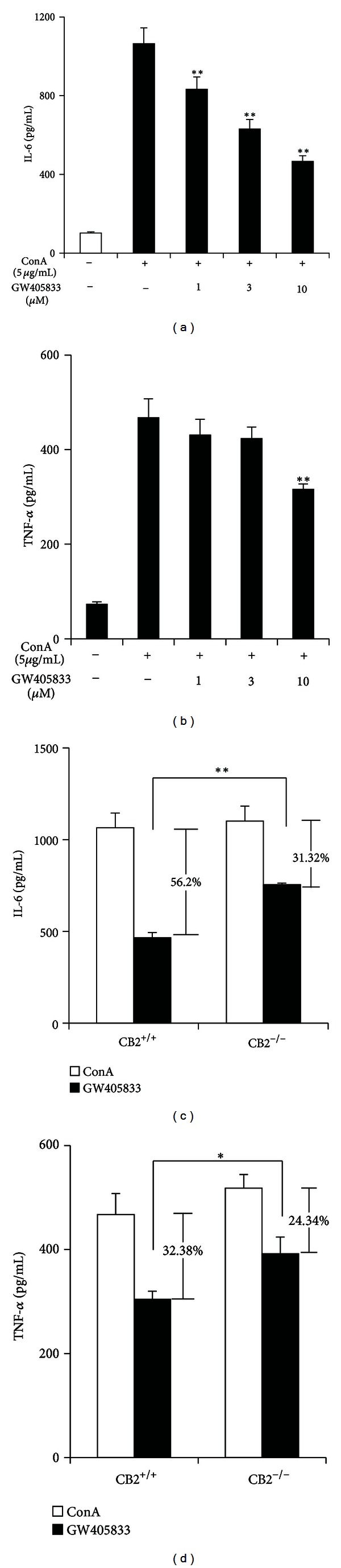
CB2R partially mediates the inhibitory effects of GW405833 on ConA-induced TNF-*α* and IL-6 production in splenocytes. (a)-(b) Splenocytes isolated from CB2R^+/+^ mice were treated with vehicle or ConA (5 *μ*g/mL) in the absence or presence of GW405833 (1–10 *μ*M) for 24 h. The levels of IL-6 and TNF-*α* in the supernatants were detected by ELISA. Data are means ± SD (*n* = 3), *P < 0.05, and **P < 0.01 versus ConA. (c)-(d) Splenocytes isolated from CB2R^+/+^ and CB2R^−/−^ mice were treated with ConA (5 *μ*g/mL) in the absence or presence of GW405833 (10 *μ*M) for 24 h. The levels of IL-6 and TNF-*α* in the supernatants were detected by ELISA. Data are means ± SD (*n* = 3), *P < 0.05, and **P < 0.01.

We also observed the effect of GW405833 on the splenocytes release of Th1 cytokine TNF-*α* and Th2 cytokine IL-6. GW405833 significantly decreased the capacity of ConA-stimulated splenocytes to release TNF-*α* and IL-6 in a dose-dependent manner (Figures 3(a) and 3(b)). However, this role was partially blocked when CB2R was knocked out. As shown in Figure 3(c), the inhibition ratio of GW405833 (10 *μ*M) on ConA-triggered IL-6 release of splenocytes was reduced from 56.2% to 31.32%. The inhibitory action of GW405833 on TNF-*α* production was reduced modestly but significantly by knockout of CB2R (32.38% to 24.34%, Figure 3(d)), indicating that CB2R partially mediated the inhibitory effects of GW405833 on cytokines release of splenocytes.

### 3.4. CB2R Is Critical for the Inhibitory Role of GW405833 on LPS-Triggered IL-6, TNF-*α*, and HMGB1 Production in Macrophages

**Figure 4:**
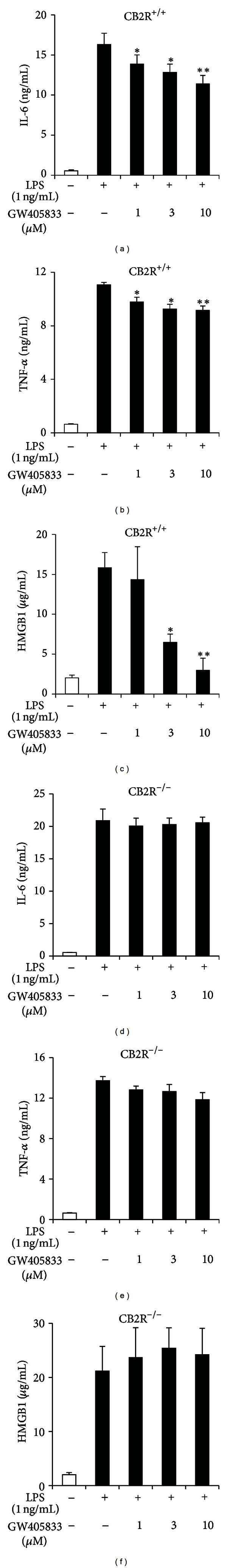
CB2R is critical for the inhibitory role of GW405833 on LPS-triggered IL-6, TNF-*α*, and HMGB1 production in macrophages. (a)–(c) Peritoneal macrophages isolated from CB2R^+/+^ mice were treated with vehicle or LPS (1 ng/mL) in the absence or presence of GW405833 (1–10 *μ*M) for 24 h. The levels of IL-6, TNF-*α*, and HMGB1 in the supernatants were detected by ELISA. (d)–(f) Peritoneal macrophages isolated from CB2R^−/−^ mice were treated as described in (a)–(c). The levels of IL-6, TNF-*α*, and HMGB1 in the supernatants were detected by ELISA. Data are means ± SD (*n* = 3), *P < 0.05, and **P < 0.01 versus LPS.

We next examined the effect of GW405833 on the macrophages activation. GW405833 dose-dependently inhibits the production of IL-6, TNF-*α*, and HMGB1 in LPS-triggered peritoneal macrophages from CB2R^+/+^ mice (Figures 4(a)–4(c)), with remarkable effect in IL-6 and HMGB1 production but a weaker one in TNF-*α* production. However, these inhibitions were completely abolished in peritoneal macrophages from CB2R^−/−^ mice (Figures 4(d)–4(f)).

### 3.5. GW405833 Inhibits LPS-Triggered Signal Pathway in Macrophages

**Figure 5:**
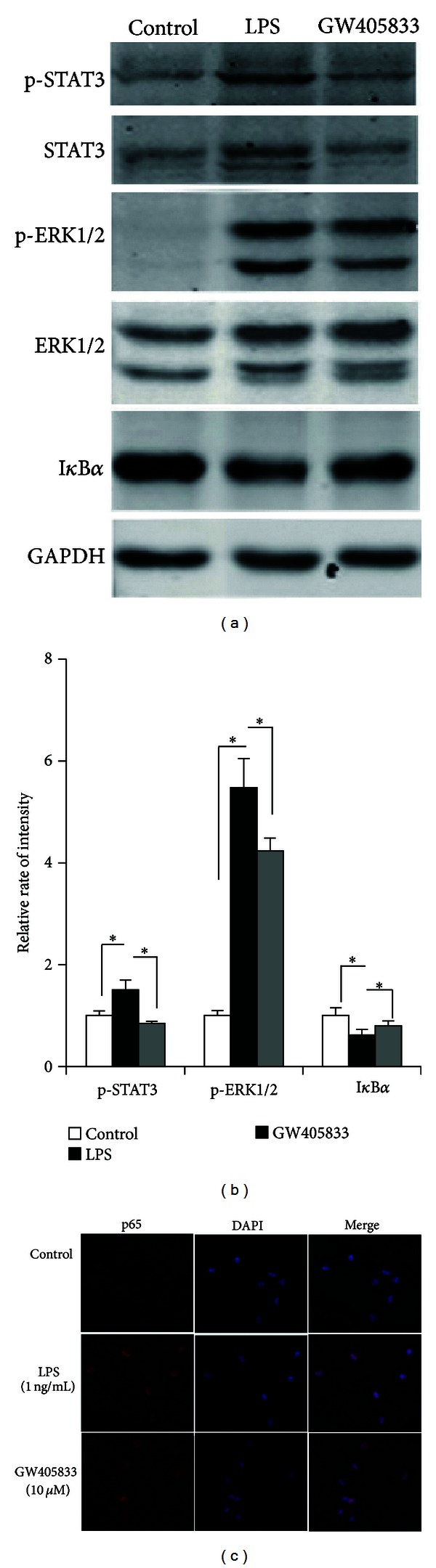
GW405833 inhibits LPS-triggered signal pathway in macrophages. (a) Peritoneal macrophages isolated from C57BL/6 mice were treated with vehicle or LPS (1 ng/mL) in the absence or presence of GW405833 (10 *μ*M) for 30 min. STAT3, phosphorylated-STAT3, ERK1/2, phosphorylated- ERK1/2, and I*κ*B*α* were detected by Western blotting. Data are representative of three independent experiments. (b), Quantification of the result of (a). p-STAT3 expression was normalized to STAT3, p-ERK1/2 was normalized to ERK1/2, and I*κ*B*α* was normalized to GAPDH. Data are means ± SD, *P < 0.05, and **P < 0.01. (c) Macrophages were treated as described in (a), p65 was detected by immunofluorescence. p65 was marked in red fluorescence and nucleus was marked with DAPI in blue. Data are representative of three independent experiments.

The stimulation of TLR4 by LPS can activate the MyD88-independent pathway resulting in the activation of NF-*κ*B and MAPK cascades [2]. The NF-*κ*B signaling pathway is critical in the pathogenesis of sepsis shock [27, 28]. Briefly, upstream signals lead to phosphorylation and ubiquitin-dependent degradation of I*κ*B*α* or I*κ*B*β* and translocation of p65-related dimmers into the nucleus followed by subsequent gene transcription. There also are reports that ERK1/2 [29–31] and STAT3 [32, 33] as upstream regulator participating in the activation of NF-*κ*B signaling pathway in sepsis. We therefore further explored the effect of CB2R activation on the LPS/TLR4 signal pathway. Classically, LPS (1 ng/mL) triggered phosphorylation of STAT3 and ERK1/2, induced the degradation of I*κ*B*α*, and promoted the translocation of NF-*κ*B p65 from the cytoplasm into the nucleus. GW405833 (10 *μ*M) treatment inhibited these processes (Figure 5).

## 4. Discussion

Mobility in sepsis was still a main problem in clinical therapy. Study emphasis will be put on the drugs which can decrease the death in patients with sepsis. LPS was a toxic component of the outer membrane of gram-negative bacteria and high dose of LPS challenge in animal can induce a rapid response which resembles septic shock in clinical [34]. Here, we evaluated the role of CB2R in LPS-induced acute experimental sepsis model. These studies use a genetic loss and pharmacological gain of CB2R to suggest that CB2R provide a protective role in response to sepsis, indicating that the CB2R represents a possible therapeutic target for the treatment of sepsis.

Previous studies showed that the CB2R appears to produce both immunoenhancing and immunosuppressing effects during sepsis depending upon the cell type examined and severity of injury inflicted [14–17]. Our studies presented here showed that CB2R activation produced immunosuppressing effects no matter in LPS-triggered macrophages or ConA-triggered splenocytes, which is consistent with a previous report that the inhibition of LPS mediated NO release by WIN55212 was mediated by CBR2 in murine macrophages [35]. Furthermore, no matter in the lethal dose of LPS-induced septic shock or in the low dose of LPS-induced endotoxemia, CB2R activation by GW405833 showed a protective role, which increased the survival rate and decreased the serum proinflammatory cytokines levels.

It is widely reported that the endocannabinoid system is upregulated during sepsis, although a recent study reported that LPS downregulated CB2R expression in peritoneal macrophages [36]. In the sera of patients and animals suffering from sepsis, the concentrations of endogenous ligands of CBR (2-AG and anandamide) were elevated [37, 38]. LPS treatment caused a time dependent increase in CB2R expression in macrophages [39]. Several studies reported that endocannabinoids can modulate the release of proinflammatory mediators via CB2R-related pathways. Particularly, 2-AG inhibits cytokine production in LPS-treated murine macrophages and IL-2 secretion in activated murine splenocytes. Anandamide inhibits lymphocyte proliferation and induces cell death by apoptosis [40–42]. Our studies found that deletion of CB2R resulted in being more vulnerable to death after LPS challenge, indicating that endocannabinoids system might mediate anti-inflammatory actions through CB2R.

Macrophages serve as the first line of defense to invading pathogens. Proinflammatory cytokines such as TNF-*α*, IL-6, and HMGB1, as released from macrophages, further augment systemic inflammation [23, 43, 44]. Cells of adaptive immune system, such as naïve T cells, proliferate to generate effecter cells, which in turn liberate distinct cytokine profiles [45]. Our results demonstrated that CB2R is essential for the inhibitory role GW405833 in the production of proinflammatory cytokines in LPS-triggered macrophages but just partially mediates role of GW405833 in splenocytes proliferation and cytokines release. Reasons for this phenomenon may result from the expression of CB2R in macrophages being more abundant than that in T cells [9, 10], and also provide a clue that the possibility of the existence of other subtype cannabinoid receptors in T cells. So far, there are a few candidates reported previously, such as transient receptor potential vanilloid 1 (TRPV1) [46], orphan receptor GPR55 [47, 48], and marine cyanobacterial fatty acid amides [49], which might mediate the role of GW405833 besides CB2R in splenocytes. Anyway, it cannot be denied that CB2R is the predominant mediator for the function of GW405833 in sepsis, and compared to T cells, macrophages may contribute more in the cellular target of this process.

The CB2R was reported to signal through a G-protein coupled receptor linked to a G*α*i protein, which reduces intracellular cAMP levels by decreasing adenylyl cyclase activity [50]. In the present study, we found that GW405833 could attenuate the LPS-triggered phosphorylation of ERK1/2 and STAT3 and block LPS-induced degradation of I*κ*B*α* and translocation of p65 in peritoneal macrophages, suggesting possible crosstalk between TLR4 signal pathway and cAMP pathway. In addition, Johammes Tschop reported that in CLP-treated CB2R^−/−^ mice, p38 MAPK activation is decreased, while CB2R agonist increases p38 MAPK activation in CLP-treated CB2R^+/+^ mice [14]. Although data presented here show that the absence of CB2R is critical for sepsis, the signal pathway that mediates the protection of CB2R in sepsis is still not enough. And this issue needs further investigation.

## 5. Conclusion

Taken together, our results show that CB2R plays an important protective role in acute experimental sepsis. CB2R agonist, GW405833, could decrease mortality and proinflammatory cytokines production in LPS-challenged mice, which mainly targets T cells as well as macrophages via inhibiting LPS-trigged signal pathway. These results also indicate that CB2R is a potential therapeutic target for the treatment of sepsis.
